# A simple analytic model for predicting the wicking velocity in micropillar arrays

**DOI:** 10.1038/s41598-019-56361-7

**Published:** 2019-12-27

**Authors:** Siva Rama Krishnan, John Bal, Shawn A. Putnam

**Affiliations:** Department of Mechanical and Aerospace Engineering, University of Central Florida, Orlando, FL 32826 USA

**Keywords:** Mechanical engineering, Fluid dynamics

## Abstract

Hemiwicking is the phenomena where a liquid wets a textured surface beyond its intrinsic wetting length due to capillary action and imbibition. In this work, we derive a simple analytical model for hemiwicking in micropillar arrays. The model is based on the combined effects of capillary action dictated by interfacial and intermolecular pressures gradients within the curved liquid meniscus and fluid drag from the pillars at ultra-low Reynolds numbers ${\boldsymbol{(}}{{\bf{10}}}^{{\boldsymbol{-}}{\bf{7}}}{\boldsymbol{\lesssim }}{\bf{Re}}{\boldsymbol{\lesssim }}{{\bf{10}}}^{{\boldsymbol{-}}{\bf{3}}}{\boldsymbol{)}}$. Fluid drag is conceptualized via a critical Reynolds number: ${\bf{Re}}{\boldsymbol{=}}\frac{{{\bf{v}}}_{{\bf{0}}}{{\bf{x}}}_{{\bf{0}}}}{{\boldsymbol{\nu }}}$, where *v*_0_ corresponds to the maximum wetting speed on a flat, dry surface and *x*_0_ is the extension length of the liquid meniscus that drives the bulk fluid toward the adsorbed thin-film region. The model is validated with wicking experiments on different hemiwicking surfaces in conjunction with *v*_0_ and *x*_0_ measurements using Water ${\boldsymbol{(}}{{\bf{v}}}_{{\bf{0}}}{\boldsymbol{\approx }}{\bf{2}}\,{\bf{m}}{\boldsymbol{/}}{\bf{s}}{\boldsymbol{,}}\,{\bf{25}}\,{\boldsymbol{\mu }}{\bf{m}}{\boldsymbol{\lesssim }}{{\bf{x}}}_{{\bf{0}}}{\boldsymbol{\lesssim }}{\bf{28}}\,{\boldsymbol{\mu }}{\bf{m}}{\boldsymbol{)}}$, viscous FC-70 ${\boldsymbol{(}}{{\boldsymbol{v}}}_{{\bf{0}}}{\boldsymbol{\approx }}{\bf{0.3}}\,{\bf{m}}{\boldsymbol{/}}{\bf{s}}{\boldsymbol{,}}\,{\bf{18.6}}\,{\boldsymbol{\mu }}{\bf{m}}{\boldsymbol{\lesssim }}{{\boldsymbol{x}}}_{{\bf{0}}}{\boldsymbol{\lesssim }}{\bf{38.6}}\,{\boldsymbol{\mu }}{\bf{m}}{\boldsymbol{)}}$ and lower viscosity Ethanol ${\boldsymbol{(}}{{\boldsymbol{v}}}_{{\bf{0}}}{\boldsymbol{\approx }}{\bf{1.2}}\,{\bf{m}}{\boldsymbol{/}}{\bf{s}}{\boldsymbol{,}}\,{\bf{11.8}}\,{\boldsymbol{\mu }}{\bf{m}}{\boldsymbol{\lesssim }}{{\bf{x}}}_{{\bf{0}}}{\boldsymbol{\lesssim }}{\bf{33.3}}\,{\boldsymbol{\mu }}{\bf{m}}{\boldsymbol{)}}$.

## Introduction

Heat pipes and related devices based on capillary, thermocapillary, chemo-capillary flows are finding recognition in a wide variety of applications ranging from the cooling of microprocessors in satellites and cell phones to the thermoregulation of batteries, cryogenic sensors, and geodynamic processes^1^ to even the engineering of synthetic tissues in physiology^2^. In regard to heat mitigation devices, the recent focus has turned to chemically and mechanically patterning surfaces at the micro- and nano-scales for enhancing thin-film evaporation and condensation^3^. These textured surfaces are not only attractive because serve as fluid pump with no moving parts, but also because they can inhibit wall dryout at near critical heat flux (CHF) conditions by retaining the liquid coolant on the heated surfaces^4^. However, while capillary action due to physical, thermal, and chemical heterogeneities has provided many new opportunities for a myriad of technologies, further advances are limited by current lack of understanding of the flow-fields and phase change phenomena at high-gradients and the spatiotemporal characteristics of the intermolecular interactions at solid-liquid-vapor interfaces^5^.

In what follows, we provide a simple theoretical framework for predicting the wicking velocity in micropillar arrays with comparisons to experimental data. The resulting analytical model is based on the combined effects of fluid drag from the pillars at Reynold’s numbers constituting creeping (Stokes) flow and capillary action dictated by intermolecular forces and interfacial pressures within the curved thickness profile of the liquid meniscus. In regard to the latter, we show that the combined effects of surface energy, disjoining pressure, and Laplace pressure gradients can be encapsulated in terms of a simple, measurable length-scale referred to as the meniscus extension (*x*_0_)^6^. The meniscus extension describes the extension length of the wicking front and effectively drives the fluid flow at a rate ∝1/*x*_0_. Hence, this work provides a fundamental approach to predicting hemiwicking flow dynamics by stressing the role of the meniscus extension.

Hemiwicking samples were fabricated in Center of Nanophase Materials Sciences (CNMS) at Oak Ridge National Laboratory. Different configurations of skewed lipophilic micro-pillar arrays were fabricated by first depositing cylindrical pillar structures on Si using the Nanoscribe Pro GT laser lithography system. Then, the pillared substrates were coated with ≈20 nm of SiO_2_ to ensure a hydrophilic surface using the Oxford FlexAL Plasma Atomic Laser Deposition System. SEM was performed on samples to ensure sample integrity. Water, ethanol and FC-70 are used as the wicking media. Water is chosen for experiments to understand the Cassie-Wenzel-Hemiwicking state transitions, owing to its partial-wetting nature on SiO_2_ substrate. These wetting transitions are highly dependent on the micropillar configuration and arrangement^7,8^. Ethanol serves as moderately low viscosity and low-density fluid, whereas FC-70 is a highly viscous, high density fluid. Moreover, FC-70 has unique characteristics associated with its immiscibility – limiting the removal/desorption of absorbed gases, water, hydrocarbons, and other debris from the hemiwicking surfaces during repeated experiments in ambient environments. This fluid combination also facilitates assessment based on dissimilar interfacial adhesion forces^9^.

**Figure 1:**
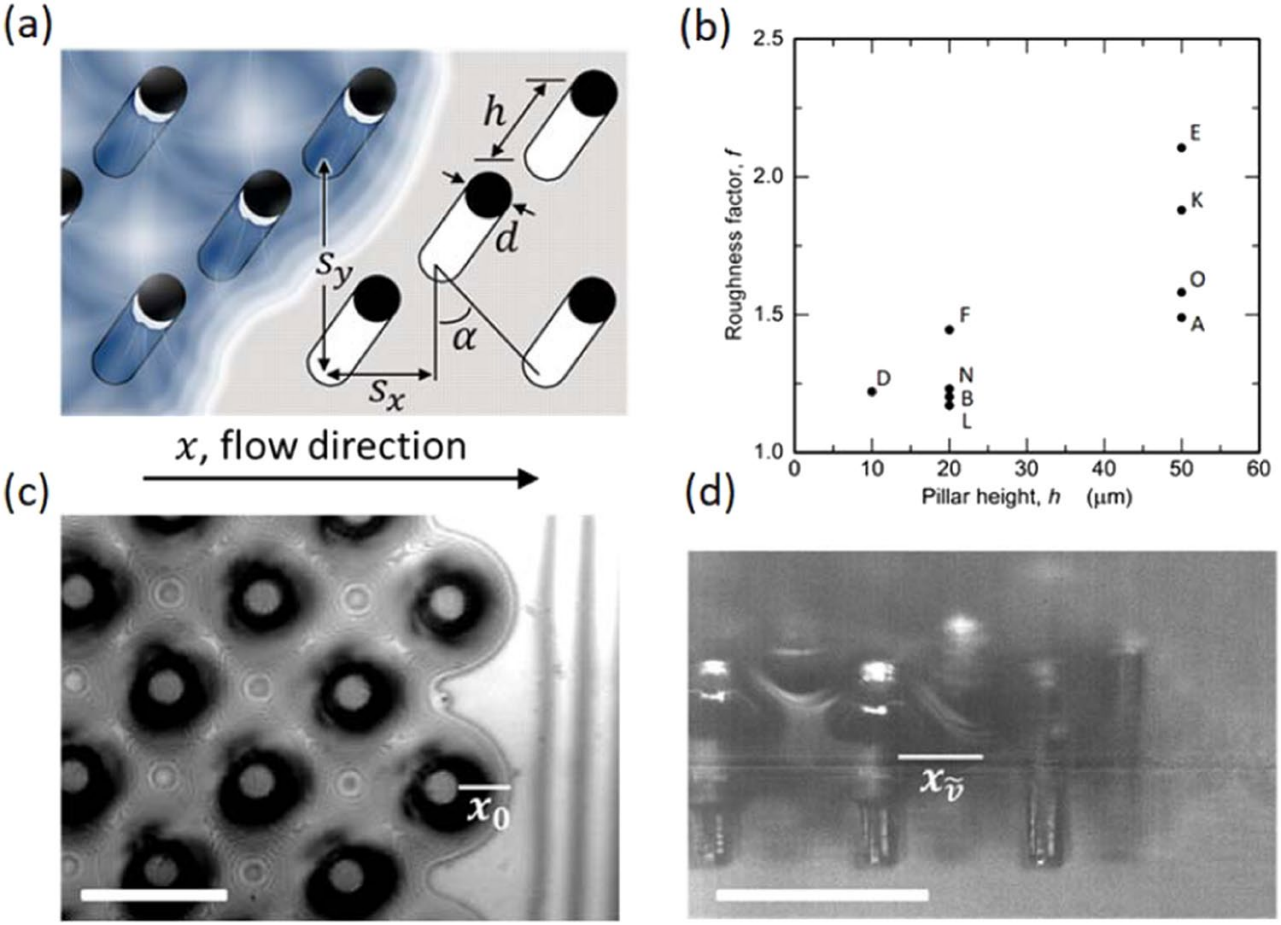
**(a)** Schematic of the micro-pillar array structure and geometry. **(b)** Scatter plot for the hemiwicking samples studied in terms of the dimensionless roughness factor (*f*) and the pillar height (*h*). **(c)** Top-view camera image showing the interferometry fringes and corresponding meniscus extension (*x*_0_) of the wicking front (scale bar: 60 μm, sample: D, λ = 656 nm, *x*_0_ = 18.6 μm). **(d)** Side-view high-speed camera image of the propagating wicking front – see Supplemental Video (scale bar: 100 μm, sample: E, ${x}_{\tilde{v}}=34.8\,\mu {\rm{m}}$).

The micro-pillar arrays are characterized by 5 independent variables (Fig. 1(a)): pillar diameter (*d*), pillar height (*h*), and pillar placement geometry based on the orientation directions (*s*_*x*_, *s*_*y*_) and the skew angle (*α*). A dimensionless roughness factor (f) is used to define the ratio of actual area to the projected surface area of the pillar array:

1 $$f=1+\frac{\pi hd}{{s}_{x}{s}_{y}}.$$

For this study, roughness factors ranged from 1.23 to 2.11 (Fig. 1(b)). The skewness angles of pillar arrays are either α = 30° or α = 45°, where α = 30° for samples (K, L) and α = 45° for all other samples.

The liquid meniscus profiles and wicking speeds are measured by interferometry and high-speed videography. Figure 1(c) provides an interferometry image of a fully-wetted hemiwicking sample (sample D, Fig. 1(b)). The meniscus extension (*x*_0_) – annotated in Fig. 1(c) at the downstream end of the pillar array – describes the extension length of a curved liquid thin-film (meniscus) that spans from a region of macroscopic fluid thickness (i.e., the bulk fluid adjacent to the wetted pillars of thickness *δ* (*x*) ≫ 1 μm) to the region of nanoscopic fluid thickness (i.e., the ultra-thin region of absorbed fluid, 10 nm $\lesssim \,{\delta }_{0}\,\lesssim$ 100 nm). Along with characterization of *x*_0_, interferometry fringe analysis facilitates spatiotemporal characterization of the meniscus curvature profiles for a variety of different fluid/sample systems^10^. In regards to the spatiotemporal evolution liquid meniscus, Fig. 1(d) provides a side-view snapshot of the wicking front during propagation on another sample (sample E, Fig. 1(b)). Hence, the meniscus length $({x}_{\tilde{v}})$ annotated in Fig. 1(d) corresponds to a spatiotemporal meniscus extension, where the instantaneous velocity of wicking front is observed to follow: $\tilde{v}\propto 1/{x}_{\tilde{v}}$. Due to the dynamic characteristics of both $\tilde{v}$ and ${x}_{\tilde{v}}$, we report the average velocity (*v*) over a pillar separation (*s*) at a given propagation length (*L*) and then correlate *v* (*L*) with the corresponding *x*_0_ measured for the different hemiwicking sample/fluid combinations. Relative to previous reports, we use this meniscus extension length (rather than a geometrical wicking structure ratio – e.g., *d*/*s*)^11,12^ to quantify the fluid wetting/wicking behavior. We note that within this transitional ‘thin-film’ (or meniscus extension) region maximum (1) evaporation rates, (2) disjoining pressure gradients, and (3) hydrostatic pressure gradients are found^13,14^.

Our initial hemiwicking experiments focused on lateral wicking with Ethanol and FC70 droplets (≈2 mm in diameter). To initiate wicking, the fluid droplets were placed at the starting end of the pillar array using a syringe needle. Then, wicking videos were recorded using a Phantom v12.1 high-speed visible camera. For example, the recorded wicking velocities ranged between 0.42–28.8 mm/sec with ethanol and 0.01–1 mm/sec for FC70. These wicking velocities correspond to Reynolds number flow regimes between 10^−3^ and 10^−7^.

**Figure 2:**
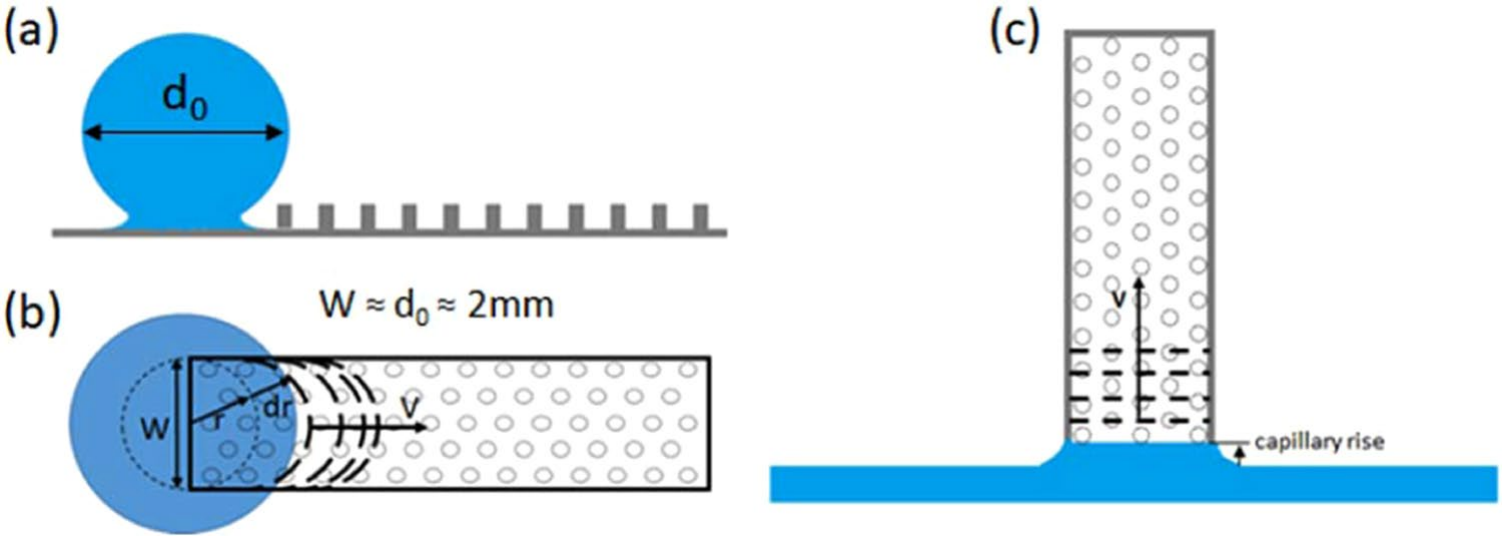
Schematic illustration of the early stages of hemiwicking ‘front’ propagation for both **(a,b)** horizontal (or lateral) wicking from a fluid (sessile) droplet reservoir and **(c)** vertical wicking from a flat pool reservoir.

We studied hemiwicking with both sessile droplets (Fig. 2(a,b) and free-surface pools (Fig. 2(c)). The sessile droplet studies used 2 mm in diameter droplets dispensed from a syringe needle at ultra-low Weber numbers (We < 0.22). The corresponding Bond numbers were Bo < 0.2; therefore, gravity effects can be ignored. For these lateral hemiwicking experiments the meniscus front radially expands (due to free energy minimization – not gravity) on both the flat substrate and inside first rows of pillars (Fig. 2(b)). The diffusion within this time-scale is dominated by wetting dynamics of the droplet, resulting in early-stage hemiwicking with a smooth circular velocity profile. However, in the later-stages of lateral hemiwicking from a sessile droplet, the velocity profile transitions from circular to parabolic to relatively flat (or uniform) across the width of the pillar-array. For vertical wicking from a free-surface pool, the leading-edge of the meniscus initially wets the first row of pillars (due to hemiwicking imbibition) with a relatively flat velocity profile across the width of the pillar-array (Fig. 2(c)). For vertical wicking, the wicking velocities ranged between 3–8 mm/s, 1–11 mm/s, and 0.1–0.8 mm/s for water, ethanol, and FC-70, respectively. We observe that the wicking velocity from free-surface pool is approximately a factor of *π* slower than that for lateral wicking from a sessile droplet. This factor of *π* difference is accounted for later in the manuscript as a scaling for the curvature of the advancing meniscus front.

While the wetting model by Wenzel^15^ provides a reasonable account of physics driving the wetting behavior within the equilibrium pinning length, the wetting characteristics with heterogeneous pillar-substrate configurations (or chemically heterogeneous surfaces) are too complex to be described by such a general model due to the disjoining pressure and other long range intermolecular forces^16^. With this said, the bulk fluid near the wetted pillars do not contribute significantly to wicking because the bulk fluid is thick (*δ* ≫ *δ*_0_) leading to negligible long-range intermolecular forces and disjoining pressure $(\Pi )$ gradients (e.g., $d\Pi /dx\propto 1/{\delta }^{4}$ for *δ* ≫ 1 μm). Whereas, at the edge of the meniscus extension (x_0_), the wicking dynamics are dominated by intermolecular liquid-substrate interactions for sufficiently thin films. Therefore, inclusion of *x*_0_ is necessary in modeling wicking front dynamics. For simplicity, we relate the driving force for wicking (*F*_*w*_) to the Laplace pressure, which also depends on the meniscus curvature that scales as *h*/*x*_0_. While previous reports^17–20^ capture the dynamics of imbibition, the effects of meniscus curvature and adsorbed thin-film thickness (e.g., Laplace and disjoining pressures, respectively) were not identified. For example, the meniscus extension (*x*_0_) has not been previously used as a scale for the wicking velocity and no prior correlation exists between the experimental values of *x*_0_ and $h{s}_{y}/\pi d$. This over-simplification of the wetting at macroscopic length-scales ignores the role of intermolecular bonding forces, disjoining pressures, and gradients therein.

While the driving force for the hemiwicking flow originates from the unwetted surface area, the resistance to the flow arises primarily from the viscous drag over the submerged pillars. Inside the bulk fluid, the pressure gradients can be neglected $(dP/dx\approx 0)$, which also satisfies mass continuity for one dimensional flow. However, within the curved meniscus extension region, the pressure gradients cannot be neglected and should be compared to the pressure discontinuity (jump) that exists at a liquid-vapor interface. Moreover, for the wicking front to advance, it must overcome this pressure discontinuity. With these considerations, wicking flow can be modeled by a simple displacement-work method, noting that the maximum wicking velocity for this pressure-driven flow should be limited by the maximum rate for free energy minimization. The pressure jump, or the Laplace pressure, is a product of the surface tension coefficient and the meniscus curvature. Hence, the Laplace pressure follows as

2 $${P}_{{\rm{Laplace}}}=\frac{\gamma h}{{x}_{0}^{2}}.$$

The product of magnitude of Laplace pressure and incremental volume change (due to wicking front propagation) gives a scale for the work done in this wetting process: $P\Delta V={P}_{{\rm{Laplace}}}hW\Delta L$, where *W* is the pillar array width, *h* is the pillar height, $\Delta L$ is incremental change in the spatial location of the wicking front. Ignoring the small volume of fluid menisci at the top of pillars during the propagation of the wicking front, the driving force for wicking can be expressed as:

3 $${F}_{w}=\frac{P\Delta V}{\Delta L}=\frac{\gamma W{h}^{2}}{{x}_{0}^{2}}.$$

Due to their lipophilic nature, the wetted pillars during hemiwicking are assumed to have a thin layer of stagnant fluid layer around them. To calculate drag force, we use a coefficient of drag (*C*_*d*_) and the projected pillar area normal to the flow. This simple approach accounts for hydrodynamic drag using a total drag area of ${A}_{{\rm{drag}}}={N}_{p}(hd)$, where ${N}_{{\rm{p}}}=LW/({s}_{x}{s}_{y})$ is number of submerged pillars for a wicking front that has propagated a distance *L*. In the bulk fluid, the upward capillary force at the top of the pillars closely balances the weight of the propagating front. Hence, the viscous drag from the base substrate base can be neglected in the analysis and, in result, the resisting (drag) force for wicking becomes

4 $${F}_{d}=\frac{1}{2}\rho {v}^{2}{C}_{d}\frac{(LW)(hd)}{{s}_{x}{s}_{y}}.$$

Since the flow regime follows ${{\rm{Re}}}_{d}\ll \,1$, we assume a coefficient of drag for cylindrical pillars of the form: ${C}_{d}={C}_{1}/{{\rm{Re}}}_{d}$. In support, Morison’s drag coefficient for a cylinder^21^ with creeping (Stokes) flow has the primary form ${C}_{d} \sim 4\pi /{{\rm{Re}}}_{d}$, where ${{\rm{Re}}}_{d}=\rho vd/\mu$. For creeping flow, the inertial forces must balance (no inertial flow); therefore, the drag force should be of the same order as the driving force due capillary action and imbibition. Now, scaling *F*_*w*_ with *F*_*d*_, the wicking velocity is expected to scale as:

5 $$v \sim \frac{2\gamma h{s}_{x}{s}_{y}}{{C}_{1}\mu L{x}_{0}^{2}}.$$

To compare this predicted wicking velocity to the maximum velocity that can be expected for creating a new solid-liquid interface, we measured with high-speed videography the maximum propagation speed of a fluid’s wetting front immediately after contact with a flat, dry substrate surface. Such upper-limit or critical wetting (and/or dewetting) speeds are of interest in many practical applications^22^. We note that recently Peters *et al*.^23^ and Pirat *et al*.^24^ investigated the unique regime of transitional fluid wetting in lyophobic micropillar arrays at near critical contact angles. In this unique wetting regime, the wetting front propagates via successive zippering events. The zippering velocities (*v*_zip_) reported on those substrates were as high as ${v}_{{\rm{zip}}} \sim 0.7\,{\rm{m}}/{\rm{s}}$. The maximum wetting velocity introduced in this work is directly tied to this the zipping velocity – i.e., the zipping velocity must be bound by the maximum speed for wetting (*v*_zip_ < *v*_0_). Furthermore, for unique hemiwicking structures driven by zippering effects, the wicking velocity will scales as $\propto {v}_{{\rm{zip}}}\times {l}_{{\rm{row}}}$ until bulk drag/friction effects become relevant–where *l*_row_ is the row length for the pillars tangential the flow direction and maximum wetting speeds should be expected when ${v}_{{\rm{zip}}}\approx {v}_{0}$.

Figure 2 depicts the hemiwicking front in the early stages of the wicking process for the two configurations studied in this work: (a,b) horizontal (or lateral) wicking from a sessile droplet and (c) vertical wicking from a pool of fluid. In case of wicking driven by zippering effects, the wicking front propagates with successive lateral (zippering) events that are perpendicular to the net horizontal (or vertical) wicking direction. In such cases, the zippering velocity (*v*_zip_) induces at some point a constant terminal velocity of the wicking front that is less than *v*_zip_. However, at the onset of hemiwicking, the wicking dynamics are dominated by the velocity profile (e.g., initially parabolic). Then after propagating beyond capillary length scales, the wicking front then generates a flat (or top-hat) profile. Then, flow-field breaks down from the idealized 1-D flow to 2-D flow. The present study focuses on the front velocity ignoring such 2-D flow-field effects. Hence, the proposed hemiwicking model will not be accurate for such 2-D flow regimes (i.e., those essentially equivalent to 2-D diffusion problem). In case of vertical wicking, the wicking front has a flat (or top-hat) profile from onset of suction/exposure to the fluid reservoir. Thus, at the onset of vertical hemiwicking from a flat pool reservoir, we observe that flow is initially driven by high zippering velocities/effects. Yet, these velocities are observed to be bound by *v*_0_ (not *v*_zip_).

We characterized this maximum wetting velocity (*v*_0_) for water, ethanol, and FC70 on non-structured (flat) Si wafers using different droplet diameters (1 mm and 2 mm). These *v*_0_ experiments consisted of both freely falling droplets (We < 0.22) and hanging-sessile droplets from a syringe needle. These experiments yielded ${v}_{0}^{{\rm{water}}}=2.0\pm 0.4\,{\rm{m}}/{\rm{s}}$, ${v}_{0}^{{\rm{EtOH}}}=1.24\pm 0.3\,{\rm{m}}/{\rm{s}}$, ${v}_{0}^{{\rm{FC}}70}=0.272\pm 0.035\,{\rm{m}}/{\rm{s}}$ for water, ethanol, and FC70 (respectively) on Si, where variations in droplet size and needle-droplet interactions did not influence *v*_0_ beyond the standard error indicated.

We note that these short time-scale droplet wetting experiments on a dry, free surface represent an upper-limit condition for hemiwicking flow. In this regard, we hypothesize that our scaling law (Eq. (5)) becomes exact with knowledge of the drag coefficient (*C*_*d*_ = *C*_1_/Re_*d*_) that can be coupled to a dimensionless structural (drag) factor. Therefore, the velocity scale (Eq. (5)) is refined by multiplying it by the dimensionless structural coefficient (*f* − 1) and then determining the drag coefficient (*C*_1_) from experiments. Since the hemiwicking flow is one-dimensional, we multiply Eq. (5) by (*f* − 1)^1*/*2^. Further, we note that the pillar placement orientation *s*_*y*_ in Eq. (5) should be replaced by (*s*_*y*_–*d*) to accommodate for the limiting case: *v → *0 as *s*_*y*_ → *d* and *s*_*x*_ → *d*. With these aforementioned considerations, the modified scaling law for the wicking velocity with structural drag becomes:

6 $$v \sim \frac{\gamma }{{C}_{1}\mu }\frac{h{s}_{x}({s}_{y}-d){(f-1)}^{1/2}}{L{x}_{0}^{2}}.$$

**Figure 3:**
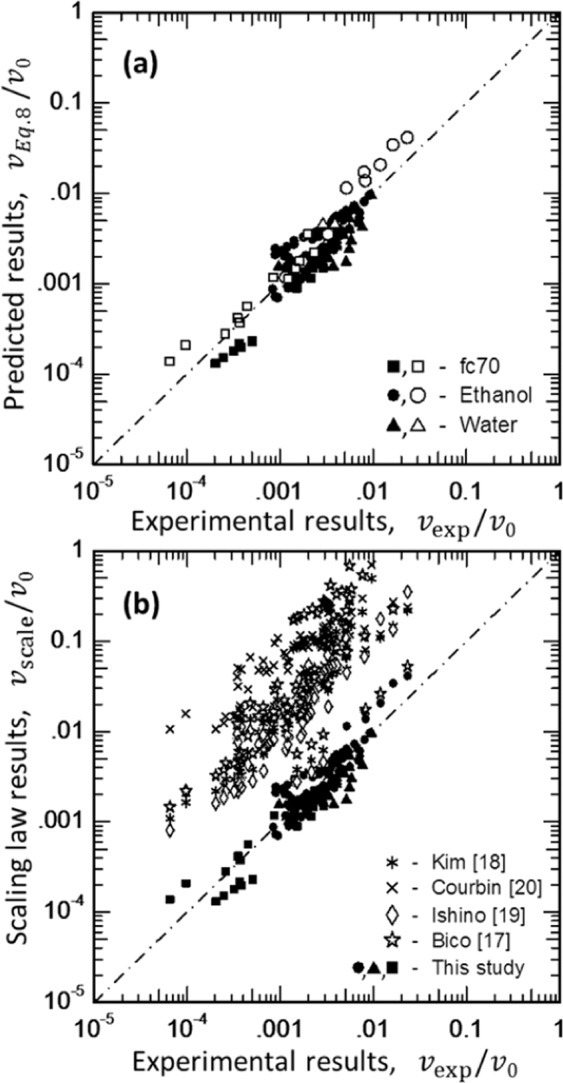
**(a)** Comparison between the predicted and measured wicking velocities. The predicted values use C_1_ = 95 in Eq. (6). The data are normalized by the maximum wetting velocity measured for droplet wetting on a flat, dry substrate (${v}_{0}^{{H}_{2}{\rm{O}}}=2.0\,{\rm{m}}/{\rm{s}}$, ${v}_{0}^{{\rm{EtOH}}}=\,1.24\,{\rm{m}}/{\rm{s}}$, ${v}_{0}^{{\rm{FC}}70}=0.27\,{\rm{m}}/{\rm{s}}$). The data are also based on measurements at different wicking lengths *L* ≈ 2, 4, and 6 mm from the fluid source, using samples A, D, F, K for FC-70, B, K, O for ethanol and, E and K for water respectively. **(b)** Data in a) revisited with additional experimental data scaling relations in the literature. The open and filled symbol data are normalized by ${v}_{0}^{{H}_{2}{\rm{O}}}=\,2.00\,{\rm{m}}/{\rm{s}}$, ${v}_{0}^{{\rm{EtOH}}}=\,1.24\,{\rm{m}}/{\rm{s}}$ and ${v}_{0}^{{\rm{FC}}70}=0.27\,{\rm{m}}/{\rm{s}}$ (respectively), where for ‘this study’ ${v}_{{\rm{scale}}}$ corresponds to Eq. (6–8) using a best fit value of *C*_1_ = 95 and for the other source data ${v}_{{\rm{scale}}}=D/2L$ with ${D}_{{\rm{Bico}}}=\frac{\gamma }{\mu }\frac{h(f-1)}{(1-\frac{\pi {d}^{2}}{4{s}_{x}^{2}})}$, ${D}_{{\rm{Ishino}}}=\frac{\gamma }{\mu }\frac{d{h}^{2}}{{s}_{x}^{2}}$, ${D}_{{\rm{C}}ourbin}=\frac{\gamma }{\mu }{s}_{x}$, and ${D}_{{\rm{K}}im}=\frac{\gamma }{\mu }\frac{h(f-1)}{[1+\frac{h}{s}(f-1)]}$.

Figure 3(a) provides data for our measured wicking velocity data in comparison to that expected (Eq. (6) with *C*_1_ = 95). To emphasize the significance of the maximum wetting velocity, the results are normalized by the measured *v*_0_ for each corresponding wicking fluid. Figure 3(b) revisits the same hemiwicking data in Fig. 3(a), but this time using the best fit value of *C*_1_ = 95. Also included in Fig. 3(b) are hemiwicking data from different sources in terms of their corresponding velocity scaling laws. For these additional literature comparisons, we also normalized the velocity data by the maximum wetting velocity (*v*_0_).

As indicated in Fig. 3(b), our scaling law for the wicking velocity (Eq. (6)) is in excellent agreement with a simple force balance theory for *C*_1_ = 95. We note that this fitted value of *C*_1_ is roughly seven times that of 4 *π*. Therefore, revisiting Eq. (6) with regard to creeping (Stokes) flow $({C}_{d}={C}_{1}/{{\rm{Re}}}_{d}\approx 4\pi /{{\rm{Re}}}_{d})$, we postulate the concept of a critical Reynolds number in terms of both the meniscus extension and maximum wetting velocity – i.e., ${{\rm{Re}}}_{{x}_{0},{v}_{0}}=\frac{{v}_{0}{x}_{0}}{\nu }$. In such a case, the flow velocity in a micro- or nano-structured array is expected to be limited (or bound) by this critical Reynolds number – noting that the effects of array structure, pillar geometry, and the fluid-substrate interactions are intrinsically coupled into both meniscus extension (*x*_0_) and the maximum speed for free surface flow (*v*_0_). Moreover, we can further define a meniscus drag coefficient in terms of this critical Reynolds number as ${C}_{d0}=95/{{\rm{Re}}}_{{v}_{0},{x}_{0}}$, which leads to a more appropriate expression for wicking velocity,

7 $$v=\frac{\gamma h{s}_{x}({s}_{y}-d){(f-1)}^{1/2}}{{C}_{d0}{{\rm{Re}}}_{{x}_{0},{v}_{0}}\mu L{x}_{0}^{2}}.$$

The validity of both Eq. (6) and Eq. (7) are demonstrated in Fig. 3(b) for roughness factors in the range of 1.17–2.11, where *C*_1_ = 95 corresponds to a respectable meniscus drag coefficient in the range of $0.6\lesssim {C}_{d0}\lesssim 5$ for both ethanol and water and 60 $\lesssim \,{C}_{d0}\,\lesssim \,185\,{\rm{for}}\,{\rm{FC}}-70$. The significantly larger meniscus drag coefficient for FC-70 is potentially associated with its immiscibility with absorbed gases, water, and hydrocarbons (which are certainly present and unavoidable in practical laboratory environments). Nevertheless, the velocity trends shown in Fig. 3(b) are consistent with work by Kim *et al*.^18^, where Eq. (6) scales with a similar trend for *C*_1_ = 25. While, the wicking velocity is found to scale directly with the pillar height, this scaling breaks down for pillars with heights much greater than or much less than the meniscus extension ($h\gg {x}_{0}$ and $h\ll {x}_{0}$, respectively). Courbin *et al*.^20^ omitted the effect of height, leading to an inaccurate prediction of wicking velocity. Bico *et al*.^17^ considered drag only from the base (substrate), while ignoring the drag from the pillars. For square micropillar arrays with pitch (*p*), the velocity scale proposed by Ishino *et al*.^19^ holds well for both $h\gg p$ and $h\ll p$. Outside these limits for $h\approx p$ and prior to the study, the velocity scale proposed by Kim *et al*.^18^ showed to correlate best with experimental data. While the velocity scale by Kim *et al*. is accurate and accounts for a dimensionless structural coefficient (*f*), in these limiting cases (as well as for $h\approx p$) their proposed velocity scale is nearly 5 times the observed value on absolute terms. There are two potential reasons for this, the scaling model by Kim *et al*. ignores the drag from pillar frontal area (which is the major source of flow resistance in our analytic model) and *ii*. Kim *et al*. mainly analyzed wicking regimes for ${{\rm{Re}}}_{d}\,\gtrapprox \,1$. In the regime ${{\rm{Re}}}_{d}\ll 1$, *C*_*d*_ grows rapidly as 1/Re_*d*_. For this report, we estimated *C*_*d*_ for ultra-low Reynolds numbers $({10}^{-7}\lesssim {{\rm{Re}}}_{d}\lesssim {10}^{-3})$, which has never (to the best of our knowledge) been quantified in the past. Also, the role of meniscus extension (*x*_0_) was completely overlooked in all previous work, which is essential for better understanding and designing wicking structures with minimal damping. While, in the surface region between two pillars, the hemiwicking flow dynamics depends mainly on the substrate’s surface energy relative to the fluid’s surface tension; viscous damping in this region may perhaps be overcome by fabricating high surface energy micro- to nano-scale surface textures to extend the ‘foot’ of meniscus^25^. However, we note that the zipping events in our samples should be different from previous studies, as the majority of the prior studies used square or rectangular pillar arrays. Future studies should further investigate zipping effects for different Sx/Sy ratios with quantification of the transient meniscus extension and its coupling to the dynamic contact angle. Such detailed studies on meniscus instabilities and spatiotemporal tailoring the meniscus extension will thus be very useful in designing infinitely wicking arrays.

Multiplying the wicking velocity in Eq. (7) by the propagation distance $(Lv=L\dot{L})$, we obtain a dynamic constant associated with the spreading behavior. Alternately, we can interpret this spreading behavior in terms of a wicking front (or interfacial) diffusivity: *L*^2^ = *D*_*i*_*t* with ${D}_{i}=\frac{2\gamma h{s}_{x}({s}_{y}-d){(f-1)}^{1/2}}{95\mu {x}_{0}^{2}}$. Furthermore, a solid-liquid structure factor ‘*S*’ for wicking on heterogeneous surfaces can be defined as $S={K}^{2}/{x}_{0}^{2}$, where $K={[h{s}_{x}({s}_{y}-d){(f-1)}^{1/2}/L]}^{1/2}$ is interpreted as a surface texture coefficient. This surface texture coefficient (*K*) has the dimension of length, matching well with an alternative description as a surface roughness. Likewise, *K* is independent of the fluid properties, such that it is solely a function of the structural heterogeneities (e.g., surface roughness, pillar array placement, pillar geometry, etc.). Now, using our definition of a solid-liquid structure factor $(S={K}^{2}/{x}_{0}^{2})$, the predicted wicking velocity can be compactly written as either

8a $$v=\frac{S}{95\pi }(\frac{\gamma }{\mu }),\,{\rm{vertical}}\,{\rm{wicking}}\,({\rm{free}} \mbox{-} {\rm{surface}}\,{\rm{pool}})$$

8b $$v=\frac{S}{95}(\frac{\gamma }{\mu }),\,{\rm{lateral}}\,{\rm{wicking}}({\rm{sessile}}\,{\rm{droplet}})$$

where this solid-liquid structure factor (*S*) uniquely couples the geometric surface texture (*K*) to the intermolecular liquid-substrate interactions (*x*_0_) and the ratio, $\gamma /\mu$, represents a capillary velocity. The capillary number (Ca) in our experiments is in the range of 10^−6^ to 10^−3^, which confirms that hemiwicking flow is analogous to flow in porous media, dominated by capillary forces. The quantities $\frac{S}{95\pi }$ and $\frac{S}{95}$ in Eqs. 8(a) and 8(b) respectively are therefore Capillary numbers for hemiwicking flow. The absence of 1/*π* in Eq. 8(b)is a geometrical correction that accounts for the observed ‘initial’ curvature of the wicking front for horizontal (or lateral) wicking from sessile droplet reservoirs (Fig. 2). When a droplet wets the surface as in the lateral wicking case, the change in the interfacial energy at the triple-line follows: $\delta {\gamma }_{slv}\propto 2\pi rdr$, where *dr* is the radial extension of the contact line (Fig. 2(b)). For relative large droplet surface energies, $\gg \delta {\gamma }_{{\rm{slv}}}$, the net change in interface energy is ${\gamma }_{{\rm{slv}}}\propto {{\rm{\pi }}{\rm{r}}}^{2}$, which leads to $\mathrm{ln}\,{\gamma }_{{\rm{slv}}}\propto 2\,\mathrm{ln}\,r$ after linearizing in *r*. However, for vertical wicking from a free-surface pool, ${\gamma }_{{\rm{slv}}}\propto Wr$ (or $\mathrm{ln}\,{\gamma }_{{\rm{slv}}}\propto \,\mathrm{ln}\,r$). Since the meniscus work required for hemiwicking flow is proportional to overall contact line length, the extra work required for lateral wicking scales as a factor of π larger due to the initially curved meniscus front from a sessile droplet. Correspondingly, a flat (vertical) wicking front has a length equal to the width of the pillar array. And as discussed previously, the length of wicking front for both cases approaches *W* after hemiwicking beyond distances greater than one capillary length (i.e., $L > \sqrt{\gamma /\rho g}$). The experimental data from both lateral and vertical wicking are plotted on Fig. 3 using this factor of *π* consideration. The relative error between the experimental (*v*_*exp*_) and predicted (${v}_{{\rm{Eq}}.8}$) wicking velocity within one standard deviation – i.e. $\sigma [\frac{{v}_{exp}-{v}_{{\rm{Eq}}.8}}{{v}_{{\rm{Eq}}.8}}]=0.67$. This confirms that experimental results match relativity well with our predictions using *C*_1_ = 95, where faster wicking (or *C*_1_ values approaching C_1_ = 4*π*) can be expected with ideal systems (e.g., hemiwicking with ultra-clean surfaces and pure fluids in inert gaseous/vapor environments). We also note that the equilibrium contact angles for water, FC-70 and ethanol on the SiO_2_ coated Si substrates are 60°, 70°, and 20° (respectively), supporting that the effects of intrinsic wetting and hysteresis are mostly captured by our model without an explicit functionality. With this said, the product of the solid-liquid structure factor and fluid surface tension (i.e., *Sγ*) in Eq. 8 incorporates this the equilibrium wetting behavior. Better predictions are expected with knowledge and modeling based on a spatiotemporal meniscus extension (rather than the spatially-averaged – or pseudo-equilibrium – meniscus extension (*x*_0_) used in the work).

**Figure 4:**
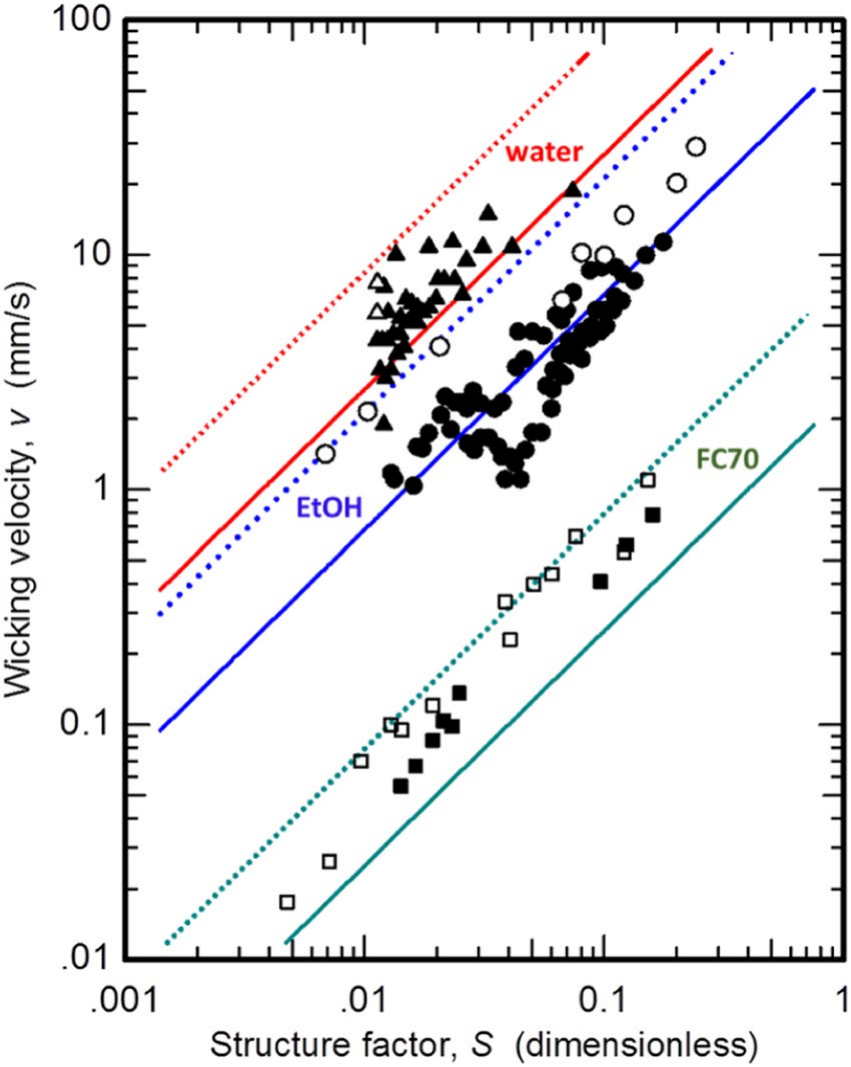
Measured (symbols) and predicted (Eq. (8), lines) wicking velocity as a function of the dimensionless solid-liquid structure factor, where the solid and dashed lines are for vertical (Eq. 8a) and lateral (Eq. 8b) hemiwicking, respectively.

Figure 4 summarizes the results of this study by comparing the predicted (lines) and measured (symbols) wicking velocity. As shown, the measured wicking velocities match closely to the predicted trends for both ethanol and FC70 over a relatively moderate range in the solid-liquid structure factor $(0.001\lesssim S\lesssim 0.25)$. This also attests that our definition of solid-liquid structure factor is a good parameter to use for predicting *v*_0_ across a broad range of Reynolds numbers $({10}^{-7}\lesssim {{\rm{Re}}}_{d}\lesssim {10}^{-3})$ in the creeping flow limit. To further support the relevance of our analytical model, Eq. (7) can be evaluated in the limiting case ($d\to 0$, $({s}_{y}-d)\to 0$, and ${s}_{x}\to d$)– i.e., the case of vanishingly close pillars such that the micropillar array is equivalent to a porous media with a pore diameter of 0.78 *d*. In this limit, we expect *x*_0_ to approach 0 (or its limiting value of ${\delta }_{0}$) and ${s}_{x}({s}_{y}-d)/{x}_{0}^{2}$ should have a non-singular value. The mean value of ${s}_{x}({s}_{y}-d)/{x}_{0}^{2}$ was computed for the range of sample systems studied. Now, assuming that this mean value should be of the same order as to that for ${s}_{x}({s}_{y}-d)/{x}_{0}^{2}$ in the limiting case, then by substituting the limiting case values for both ${s}_{x}({s}_{y}-d)/{x}_{0}^{2}$ and ${(f-1)}^{1/2}$ into the diffusivity equation we get ${L}^{2}=\frac{\gamma d}{10.86\mu }t$. Thus, we arrive at a diffusivity equation that closely follows Washburn’s popular equation for capillary flow in porous media^26^.

In conclusion, we derived a simple analytical model (Eq. (8)) that predicts the velocity of hemiwicking on substrates with micro-pillar arrays. This model is broken down into three main contributions: the fluid properties (γ/*μ*), the solid surface texture (*K*), and the liquid-substrate interactions (*x*_0_), where we defined a dimensionless structure factor $(S={K}^{2}/{x}_{0}^{2})$ to couple the heterogeneous surface texture (roughness) to the long-range intermolecular forces. The model was validated with wicking experiments of ethanol and FC70 fluids on a variety of different micro-pillar arrays. The micro-pillar arrays consisted of SiO_2_-coated cylindrical pillars on Si with center-to-center separations, diameters, heights, and corresponding surface textures (from these geometrical characteristics) that ranged within $32.3\,\mu {\rm{m}}\lesssim s\lesssim 80.1\,\mu {\rm{m}}$, $10\,\mu {\rm{m}}\lesssim d\lesssim 20\,\mu {\rm{m}}$, $10\,\mu {\rm{m}}\lesssim h\lesssim 50\,\mu {\rm{m}}$, and $0.86\,\mu {\rm{m}}\lesssim K\lesssim 5.15\,\mu {\rm{m}}$, respectively. Derivation of our analytical wicking model was highly dependent on the conceptualization and quantification of a critical (or maximum) wetting velocity (${v}_{0}^{{\rm{water}}}=2.0\pm 0.4\,{\rm{m}}/{\rm{s}}$, ${v}_{0}^{{\rm{EtOH}}}=1.24\pm 0.3\,{\rm{m}}/{\rm{s}}$, ${v}_{0}^{{\rm{FC}}70}=0.272\pm 0.035\,{\rm{m}}/{\rm{s}}$). This maximum wetting velocity is a fundamental physical property of a solid-liquid-vapor interface that is tied to the maximum rate for free energy minimization of a solid-liquid system. Combined with this maximum wetting velocity, we also showed the importance of quantifying the meniscus extension length (*x*_0_) to compactly partner the liquid thin-film profile to the wetting dynamics and intermolecular liquid-substrate interactions. Moreover, we also that *x*_0_ and *v*_0_ can be coupled together to facilitate the conceptualization of a critical Reynolds number $({{\rm{Re}}}_{{x}_{0},{v}_{0}}=\frac{{v}_{0}{x}_{0}}{\mu })$ with broader applications in many fields of science and engineering. Using this concept of a critical Reynolds number, a dynamic constant for diffusivity of the meniscus extension is found to scale as $1/{x}_{0}^{2}$. We note that the meniscus extension scales with the pillar spacing as ${x}_{0} \sim {({s}_{x}{s}_{y})}^{1/2}$ for ${x}_{0}\gg {\delta }_{0}$. In light of these observations, the meniscus extension should still be pursued with greater rigor to better understand the effects of disjoining pressure gradients due to changes in the meniscus curvature $(\propto \nabla (K/{x}_{0}))$ at chemically heterogeneous and geometrically heterogeneous solid-liquid interfaces. This meniscus curvature drives the microscopic wetting behavior of a thin film. Or, in regard to the solid-liquid structure factor $(S={K}^{2}/{x}_{0}^{2})$, to maximize the wicking velocity it is essential to increase *K* without increasing *x*_0_ at the same rate. This observation helps guide the engineering of new, spatiotemporal distributions (both geometrical and chemically) for maximizing and actively controlling the wicking velocity at speeds approaching *v*_0_, perhaps even without the need for super-lyophilic surface chemistries. We also point out that hemiwicking phenomenon can potentially be exploited to understand creeping flow dynamics^27^, which has wide range of implications at macro- to nano-scales in applications including (but not limited to) drug delivery, water harvesting in extreme environments^28^, and boiling at critical heat fluxes.

## Supplementary information

Supplementary video 1
